# Binary and ternary logic-in-memory using nanosheet feedback field-effect transistors with triple-gated structure

**DOI:** 10.1038/s41598-024-57290-w

**Published:** 2024-03-18

**Authors:** Jongseong Han, Jaemin Son, Seungho Ryu, Kyoungah Cho, Sangsig Kim

**Affiliations:** 1 Department of Semiconductor Systems Engineering, Korea University, 145 Anam-ro, Seongbuk-gu, Seoul, 02841 Republic of Korea; 2 Department of Electrical Engineering, Korea University, 145 Anam-ro, Seongbuk-gu, Seoul, 02841 Republic of Korea

**Keywords:** Electrical and electronic engineering, Applied physics

## Abstract

In this study, we demonstrate binary and ternary logic-in-memory (LIM) operations of inverters and NAND and NOR gates comprising nanosheet (NS) feedback field-effect transistors (FBFETs) with a triple-gated structure. The NS FBFETs are reconfigured in p- or n-channel modes depending on the polarity of the gate bias voltage and exhibit steep switching characteristics with an extremely low subthreshold swing of 1.08 mV dec^–1^ and a high ON/OFF current ratio of approximately 10^7^. Logic circuits consisting of NS FBFETs perform binary and ternary logic operations of the inverters and NAND and NOR gates in each circuit and store their outputs under zero-bias conditions. Therefore, NS FBFETs are promising components for next-generation LIM.

## Introduction

With the explosive growth in data volume over the past few years, efficient data processing has become a paramount consideration in digital computing systems^1–4^. Logic-in-memory (LIM) computing has been proposed as a way to reduce data processing delay and to overcome the structural limitation of von Neumann computing; computation and memory units are physically separated in von Neumann architectures, which causes the “memory wall” problem^5,6^. LIM computing systems significantly reduce energy consumption and latency by eliminating data movement between the logic and memory units^7–11^.

LIM devices, including resistive random-access memory (RAM), magnetic RAM, and ferroelectric field-effect transistors, have been widely studied to improve system performance^9,12–15^. However, most studies have focused on binary logic systems. These systems have only two distinguishable logic levels, and thereby exhibit relatively low data integration. Multivalued logic (MVL) systems with more than three discrete logic states have attracted attention as solutions for improving the information density^16–18^. MVL systems are advantageous in terms of data processing efficiency. They can process information with fewer logic operations and reduce system complexity when compared to binary logic systems^19,20^. However, it is difficult for MVL systems to completely replace conventional binary logic systems used in most computing systems^21^. Hence, a combination of binary logic and MVL circuits is required.

There have been many studies on binary or ternary LIM. In particular, resistive RAM-based LIM performs reconfigurable binary logic operations of NAND/AND, NOR/OR, and XNOR/XOR gates^22^, magnetic tunnel junction (MTJ)-based LIM performs binary logic operations of AND/NAND, OR/NOR, and XOR/XNOR gates simultaneously^23^, and MTJ-based LIM performs reconfigurable ternary logic operations of NOR/NAND gates^24^. However, they perform the binary or ternary logic operation alone, but not both the operations. In this study, binary and ternary LIM were designed using feedback field-effect transistors (FBFETs) suitable for LIM and MVL devices. FBFETs exhibit steep switching and quasi-nonvolatile characteristics owing to the positive feedback mechanism; thus, they are advantageous for the simultaneous operation of LIM and MVL^25–28^. In particular, FBFETs with a triple-gate structure can be reconfigured in p- or n-channel modes by simply adjusting the electrical signals, which provides enhanced functionality^29,30^. Therefore, we fabricated logic circuits with triple-gated FBFETs connected in the same topology as the complementary metal–oxide–semiconductor (CMOS) logic gates and investigated the binary and ternary LIM operations of the inverters and NAND and NOR gates. These FBFETs have silicon nanosheet (NS) channels with a three-dimensional gate structure to maximize gate control capability. Moreover, silicon-based NS FBFETs have high scalability and reproducibility owing to full CMOS compatibility in the fabrication process. Silicon is a widely used material in most semiconductor devices, and provides high scalability for the fabrication process to be expanded on a large scale. Moreover, silicon is highly stable, so the electrical characteristics of the device are reproducible. Therefore, compared to most non-volatile memory device-based LIMs, our silicon transistor-based LIM has many advantages in terms of the fabrication process.

## Experimental section

**Figure 1:**
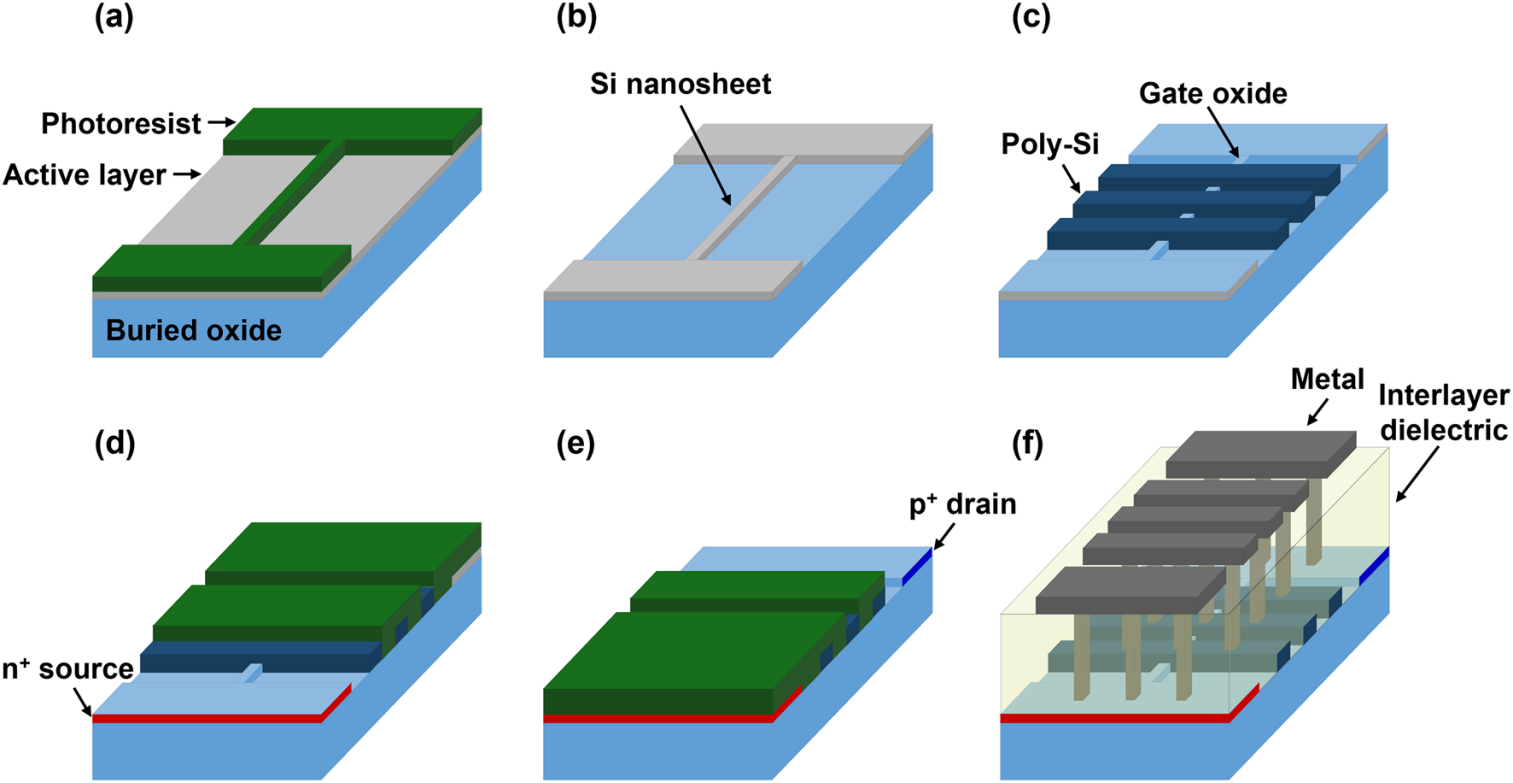
Illustration of key process steps of NS FBFET. (**a**), (**b**) Active layer patterning, (**c**) gate patterning, (**d**) source doping, (**e**) drain doping, and (**f**) contact metal patterning.

Figure 1 illustrates the key steps in the fabrication of an NS FBFET. The starting wafer was a p-type (100)-oriented silicon-on-insulator wafer with a 100-nm thick silicon active layer. After cleaning the wafer, an active layer with a width of 200 nm was formed via photolithography and dry etching (Fig. 1a,b). A 25-nm thick silicon dioxide gate dielectric layer was thermally grown on the active layer via dry oxidation. Next, a 400-nm thick polysilicon layer was deposited using low-pressure chemical vapor deposition (LPCVD). The polysilicon layer was etched with lengths of 2 μm and gaps of 1 μm via photolithography and dry etching processes to make triple gates (Fig. 1c). The source regions and two polysilicon gates adjacent to the source and drain regions were then heavily doped with P^+^ ions at a dose of 3 × 10^15^ cm^−2^ at 50 keV (Fig. 1d). Subsequently, the drain region and middle polysilicon gate were heavily doped with B^+^ ions at a dose of 3 × 10^15^ cm^−2^ at 30 keV, followed by rapid thermal annealing at 1050 °C for 30 s to activate the implanted dopants (Fig. 1e). Subsequently, a 700-nm thick interlayer dielectric layer was deposited using LPCVD-based tetraethyl orthosilicate and etched to form holes for the source, drain, and gate contacts. Finally, the Ti/TiN/Al/TiN metal stack was deposited as the source, drain, and gate electrodes, followed by an alloying process at 400 °C for 30 min (Fig. 1f).

All electrical characteristics were examined using an Agilent HP4155C semiconductor parameter analyzer and Keithley 2636B source meter at 25 °C. A cross-sectional image of the NS FBFET was captured using a JEOL JEM-2100F transmission electron microscope.

## Results and discussions

**Figure 2:**
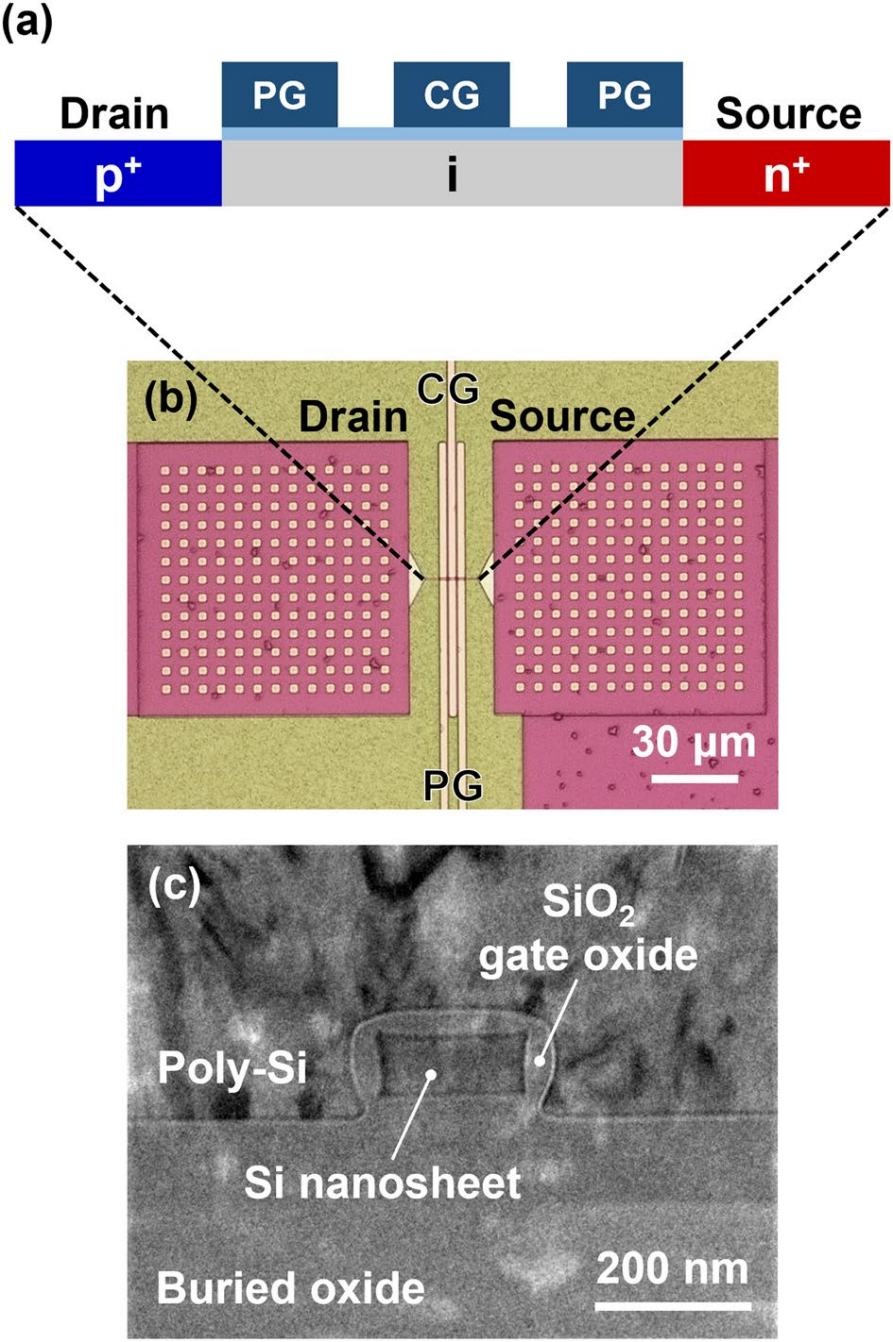
Device structure of NS FBFET. (**a**) Schematic, (**b**) optical top-view image, and (**c**) cross-sectional TEM image of fabricated NS FBFET.

Figure 2a,b shows the schematic and optical images of an NS FBFET consisting of a p^+^ drain region, an n^+^ source region, and an intrinsic region with triple gates. The intrinsic region was electrostatically doped with p- or n-type by applying a bias voltage to the gate electrodes. Among the triple gates in the intrinsic region, two electrically connected program gates (PGs) were used to determine the channel mode and one control gate (CG) was used to determine the charge carrier injection into the channel. Figure 2c shows a cross-sectional TEM image of the intrinsic region and the polysilicon gate. The cross-sectional TEM image reveals that the NS FBFET has a channel width of approximately 180 nm and a channel thickness of approximately 70 nm.

**Figure 3:**
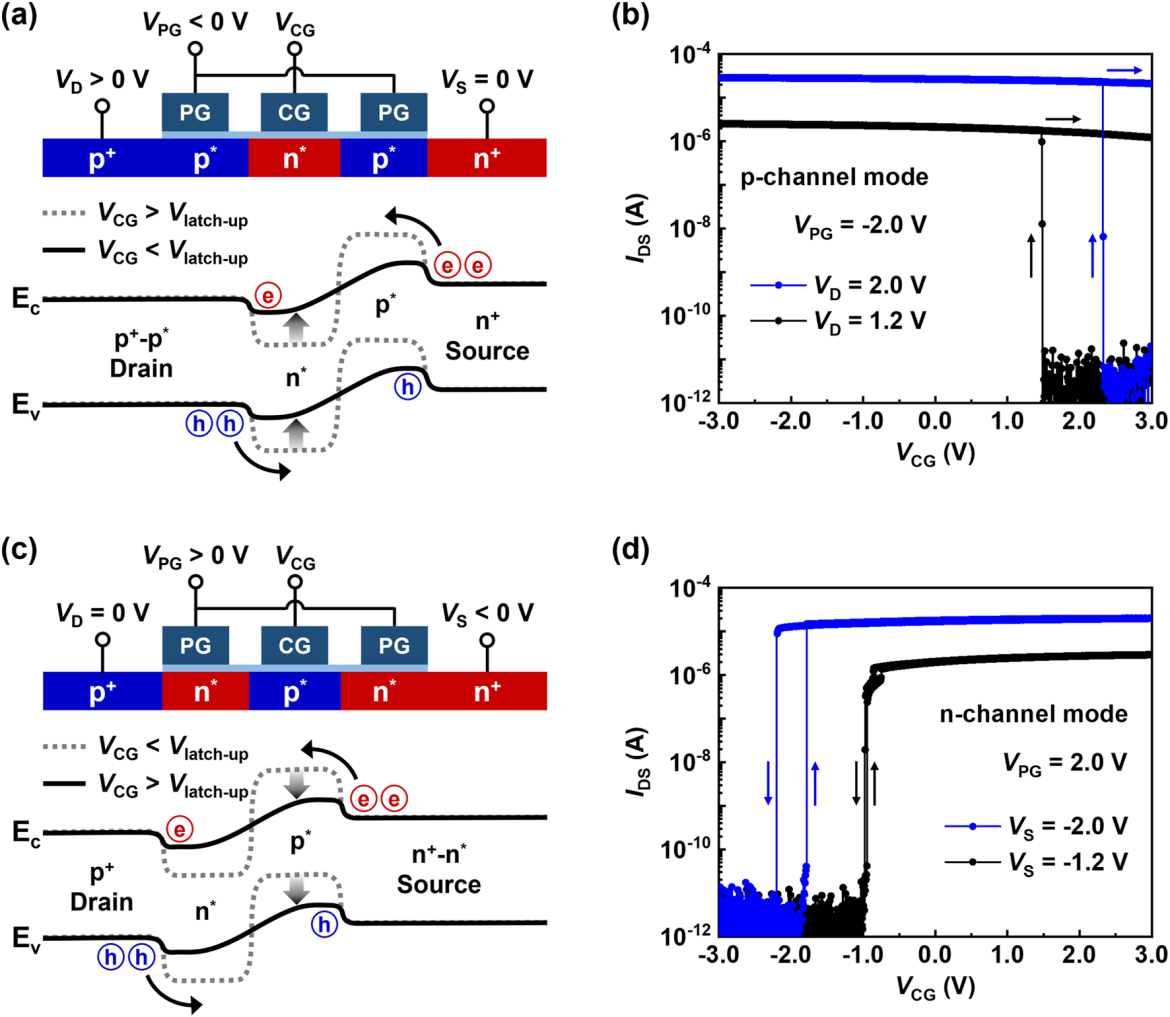
Electrical characteristics of p- and n-channel modes. (**a**) Schematic, energy band diagram, and (**b**) transfer characteristics of NS FBFET operating in a p-channel mode. (**c**) Schematic, energy band diagram, and (**d**) transfer characteristics of NS FBFET operating in n-channel mode.

The NS FBFET can be reconfigured in p- or n-channel modes based on the polarity of the PG voltage (*V*_PG_). Figure 3a shows a schematic and energy-band diagram of the NS FBFET operating in p-channel mode when a negative *V*_PG_ was applied. In the initial state of the p-channel mode, a negative *V*_PG_ creates p-type electrostatic doping (p^*^), and a positive CG voltage (*V*_CG_) creates n-type electrostatic doping (n^*^). Specifically, the energy levels of the two different p^*^ regions, generated by the same *V*_PG_, are different, as depicted by the dotted line in Fig. 3a. This is because the energy band of the p^*^ region adjacent to the drain region is effectively pinned by the drain potential owing to the large number of holes^31^. The NS FBFET has a p^+^–p^*^–n^*^–p^*^–n^+^ structure with two potential barriers that prevent charge carriers from being injected into the channel region by a positive drain voltage (*V*_D_) and electrically grounded source voltage (*V*_S_). Thereafter, as the potential barrier height is lowered by *V*_CG_, holes are injected into the channel and accumulated in the source-side potential well. This accumulation lowers the source-side potential barrier height; thus, electrons are injected from the source into the channel and accumulate in the drain-side potential well. This repetition of charge injection and accumulation triggers a positive feedback loop, resulting in the disappearance of potential barriers. The positive feedback loop allows the current to flow rapidly, which is termed as the latch-up phenomenon. The energy-band diagrams before and after the latch-up phenomenon are illustrated in Fig. 3a with dotted and solid lines, respectively.

Figure 3b exhibits the transfer characteristics of the p-channel mode at a *V*_PG_ of − 2.0 V. As *V*_CG_ is swept from 3.0 to − 3.0 V, the latch-up phenomenon occurs, and the latch-up voltage (*V*_latch-up_) varies with *V*_D_. *V*_latch-up_ changes from 1.48 to 2.33 V as *V*_D_ increases from 1.2 to 2.0 V owing to an increase in the amount of charge carriers supplied. Subsequently, as *V*_CG_ is swept from − 3.0 to 3.0 V, the transistor exhibits the hysteresis characteristics with open memory windows. This implies that electrons and holes injected into the channel generate a strong positive feedback loop, preventing the applied gate voltages from controlling the potential barriers. In other words, once the positive feedback loop is achieved, excess charge carriers are continuously injected and accumulated in the potential well in the channel region. Because excess charge carriers lower the potential barrier height in the channel region, the vanished potential barrier is not easily regenerated in the reverse *V*_CG_ sweep. Hence, when *V*_CG_ is swept enough to overcome the effects of excess charge carriers, the positive feedback loop is eliminated and the potential barrier is regenerated.

Figure 3c shows a schematic and energy band diagram of the NS FBFET in the n-channel mode. In the initial state of the n-channel mode, the NS FBFET has a p^+^–n^*^–p^*^–n^*^–n^+^ structure with a positive *V*_PG_ and negative *V*_CG_. The operating mechanism of the n-channel mode is similar to that of the p-channel mode, except that the initially injected charge carriers are electrons for negative *V*_S_. The source-side potential barrier height is lowered by the change in *V*_CG_ and electrons are injected into the channel, triggering a positive feedback loop. In Fig. 3d, the transfer characteristics of the n-channel mode at a *V*_PG_ of 2.0 V exhibit the latch-up phenomenon and changes in the *V*_latch-up_ based on *V*_S_ values. As *V*_S_ changes from − 1.2 to − 2.0 V, more electrons are injected such that *V*_latch-up_ shifts from − 0.97 to − 1.78 V. On the other hand, the transistor exhibits the latch-down phenomenon in the reverse *V*_CG_ sweep, which implies the elimination of the positive feedback loop. Compared to the p-channel mode, the n-channel mode has higher initial potential barriers owing to the work function of the polysilicon gates. Furthermore, heavily n-doped PG creates the n^*^ region, and heavily p-doped CG creates the p^*^ region^32^. Accordingly, the positive feedback loop is eliminated in the reverse *V*_CG_ sweep of the n-channel mode because the vanished potential barriers are more easily regenerated by the high initial potential barriers.

As shown in Fig. 3b,d, the NS FBFET exhibits switchable-memory characteristics due to the positive feedback mechanism. In the p-channel mode at a *V*_D_ of 2.0 V, the transistor exhibits the steep switching characteristics with an extremely low subthreshold swing (*SS*) of 1.83 mV/dec and a high *I*_ON_/*I*_OFF_ of 10^7^. Similarly, at a *V*_S_ of − 2.0 V, the n-channel mode has an *SS* of 1.08 mV/dec and an *I*_ON_/*I*_OFF_ of approximately 10^7^, which reveals the excellent characteristics as a switching transistor. Moreover, both p- and n-channel modes exhibit bistable states, which are advantageous for memory operations.

Binary and ternary LIM operations of the inverters, NAND gates, and NOR gates are performed in circuits consisting of NS FBFETs. Given the similarity between the operating mechanisms of our binary and ternary logic systems, the logic operations of both systems can be performed in a single circuit. In the binary logic system, the Input Logic 0 is defined within the range of *V*_CG_ in which the p-channel mode is turned on and the n-channel mode is turned off. Conversely, Input Logic 1 is defined within the range of *V*_CG_ in which the n-channel mode is turned on and the p-channel mode is turned off. In the ternary logic system, we use a balanced ternary number system, which is represented by three discrete logic levels of − 1, 0, and 1. Input Logics − 1 and 1 are defined in the same way as the binary logic system, and Input Logic 0 is defined within the range of *V*_CG_ in which the p- and n-channel modes are simultaneously turned on. All the LIM operations are performed with logic pulses of 1 ms and hold operations for 10 ms.

**Figure 4:**
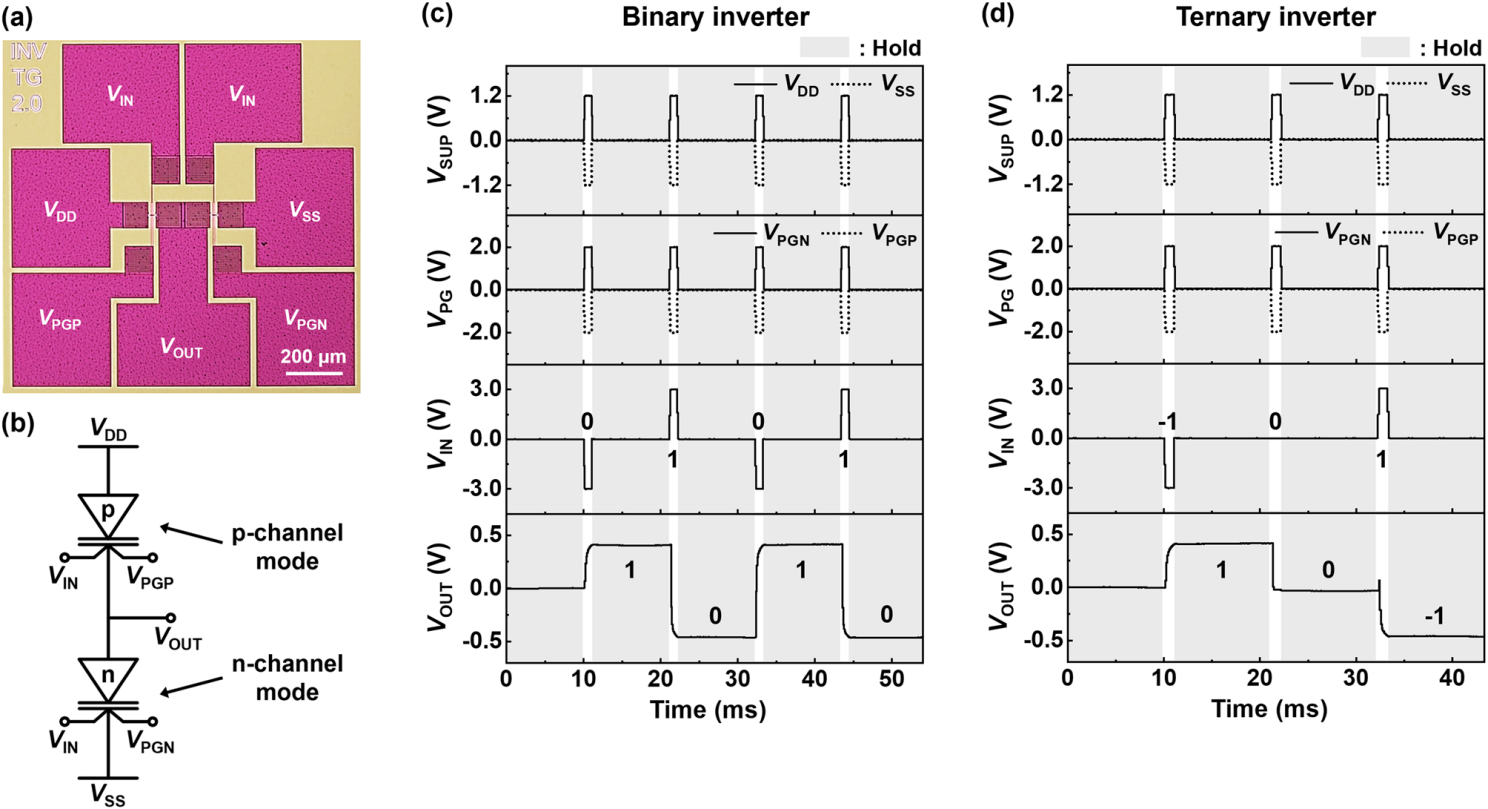
Inverting operations of NS FBFETs. (**a**) Optical top-view image and (**b**) circuit diagram of inverter using NS FBFETs. Timing diagram of (**c**) binary and (**d**) ternary inverting operation with hold operation.

Figure 4a,b shows an optical top-view image and a circuit diagram of the inverter which follows the topology of the CMOS binary inverter. The NS FBFET in the pull-up network operates in the p-channel mode with a negative *V*_PG_ (*V*_PGP_), and the transistor in the pull-down network operates in the n-channel mode with a positive *V*_PG_ (*V*_PGN_). Figure 4c shows the LIM operation of the binary inverter with power supply voltages at a *V*_DD_ of 1.2 V and at a *V*_SS_ of − 1.2 V. As an input voltage (*V*_IN_) of − 3.0 V is applied to CGs of the transistors as Input Logic 0, an output voltage (*V*_OUT_) of approximately 0.5 V is obtained as Output Logic 1 because only the transistor in the pull-up network is turned on. Conversely, as a *V*_IN_ of 3.0 V (Input Logic 1) allows the n-channel mode to turn on, a *V*_OUT_ of approximately − 0.5 V is obtained as Output Logic 0. Herein, the p-channel mode is turned off even after the positive feedback loop is achieved, unlike the single transistor characteristics. The hysteresis of the NS FBFET occurs in the DC sweep where the supply voltages are maintained. During the inverter operation, the positive feedback loop cannot be maintained consistently because the supply voltages are applied as pulses. Consequently, the p-channel mode is turned off when Input Logic 1 is applied, and the circuit comprising NS FBFETs operates as the inverter. In addition, the hold operation is performed after each logic operation, retaining the logic results under all external voltages set to 0 V. Here, each component transistor has the quasi-nonvolatile memory characteristics by storing the charge carriers in the potential well^25^. Accordingly, in the hold operation, *V*_OUT_ is determined by the accumulated charge carriers such that the output logic state is maintained under zero-bias conditions. Figure 4d shows the LIM operation of the ternary inverter. Output Logics 1 and − 1 are obtained in the same way as the binary inverter, and Output Logic 0, representing a *V*_OUT_ close to 0 V, is obtained as both the p- and n-channel modes are turned on by applying a *V*_IN_ of 0 V as Input Logic 0. For Output Logic 0, the pull-up and -down networks have similar resistances; all the component transistors are turned on. Hence, the voltage division occurs, and *V*_OUT_ is close to 0 V, which is the average value of *V*_DD_ and *V*_SS_.

**Figure 5:**
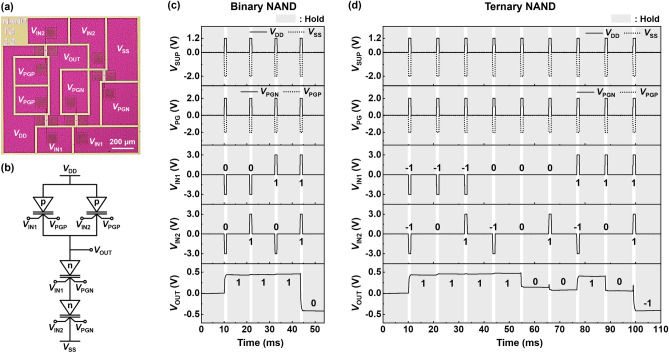
NAND gate operations of NS FBFETs. (**a**) Optical top-view image and (**b**) circuit diagram of NAND gate using NS FBFETs. Timing diagram of (**c**) binary and (**d**) ternary NAND gate operation with hold operation.

Figure 5a,b shows an optical top-view image and circuit diagram of the NAND gate, respectively, which follow the topology of the CMOS binary NAND gate. Two p-channel modes connected in parallel in the pull-up network and two n-channel modes connected in series in the pull-down network are operated with two input voltages (*V*_IN1_ and *V*_IN2_) to perform 2-input NAND gate operations. As shown in Fig. 5c, the LIM operation of the binary NAND gate is performed using four combinations of input logic: 00, 01, 10, and 11. The supplied *V*_DD_ and *V*_SS_ are 1.2 V and − 2.0 V, respectively; more voltage degradations occur in transistors connected in series rather than in parallel. As shown in the timing diagram, when either or both *V*_IN1_ and *V*_IN2_ are applied as Input Logic 0, *V*_OUT_ is obtained as Output Logic 1. Furthermore, *V*_OUT_ is connected to *V*_DD_ by the pull-up network in which the p-channel modes are connected in parallel. Additionally, when both *V*_IN1_ and *V*_IN2_ are applied as Input Logic 1, only the n-channel modes are turned on such that *V*_OUT_ is obtained as Output Logic 0.

Figure 5d shows the LIM operation of the ternary NAND gate with nine combinations. Output Logics − 1 and 1 can be obtained in a similar way to the binary NAND, even with the addition of a third intermediate logic level. For example, as *V*_IN1_ of Input Logic − 1 and *V*_IN2_ of Input Logic 0 are applied to the component transistors, all the p-channel modes are turned on and only one n-channel mode is turned on; *V*_OUT_ is connected to *V*_DD_ by the pull-up network. In the case of Output Logic 0, *V*_OUT_ is connected to *V*_DD_ and *V*_SS_ because all n-channel modes are turned on, and one or two p-channel modes are turned on. Accordingly, a *V*_OUT_ of approximately 0 V is obtained by voltage division.

**Figure 6:**
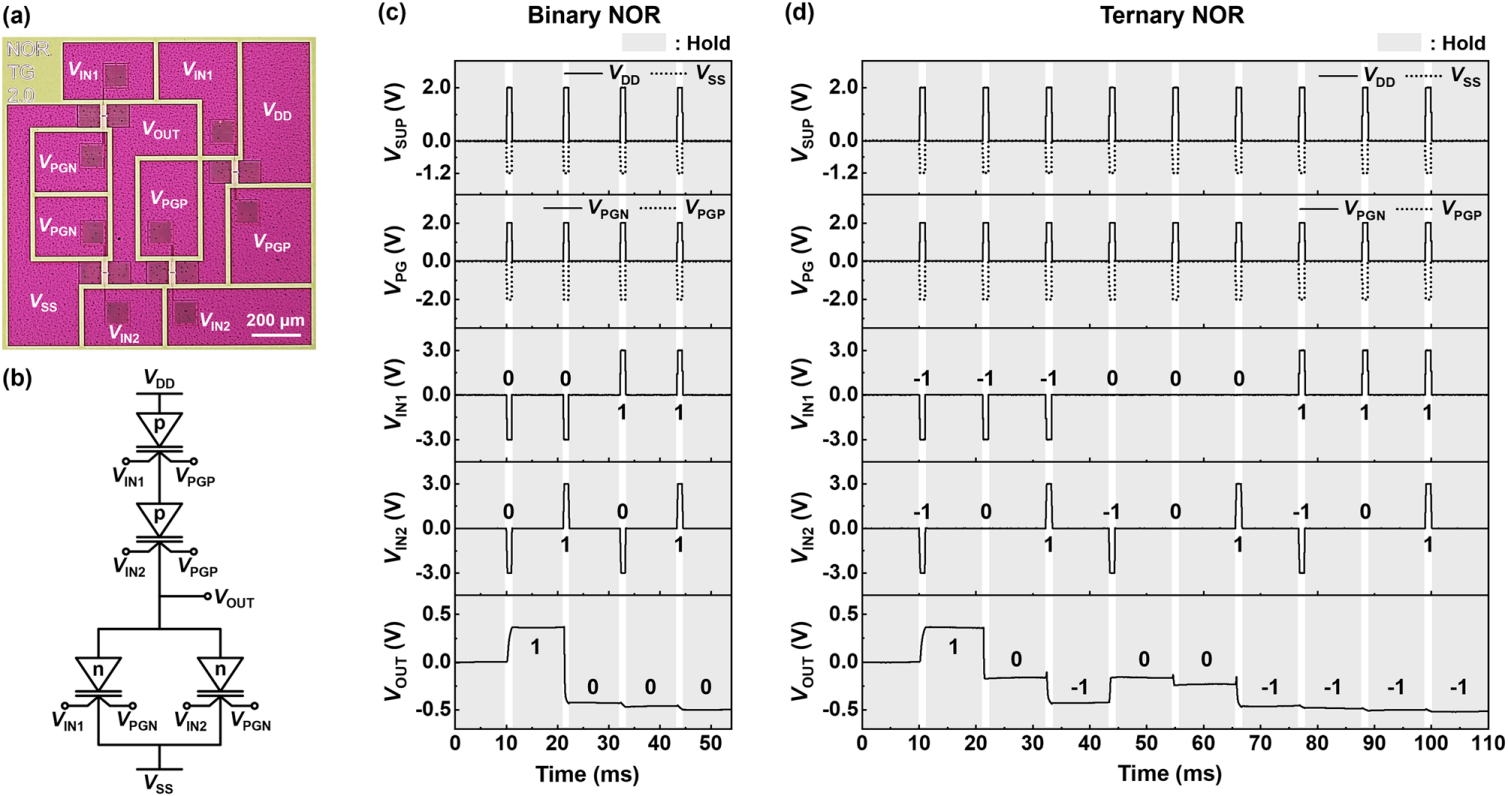
NOR gate operations of NS FBFETs. (**a**) Optical top-view image and (**b**) circuit diagram of NOR gate using NS FBFETs. Timing diagram of (**c**) binary and (**d**) ternary NOR gate operation with hold operation.

Next, Fig. 6a,b represents an optical top-view image and circuit diagram of the NOR gate, respectively. Two p-channel modes are connected in series in the pull-up network, and two n-channel modes are connected in parallel in the pull-down network. Figure 6c illustrates the LIM operation of a binary NOR gate. The component transistors of the pull-up (pull-down) network are connected in series (parallel). Hence, a *V*_DD_ of 2.0 V and *V*_SS_ of − 1.2 V are supplied. When either or both *V*_IN1_ and *V*_IN2_ are applied as Input Logic 1, Output Logic 0 is obtained; the n-channel (p-channel) mode is turned on (off). Conversely, when both *V*_IN1_ and *V*_IN2_ are applied as Input Logic 0, Output Logic 1 is obtained; only the p-channel modes are turned on. Moreover, the LIM operation of the ternary NOR gate is shown in Fig. 6d. For Output Logics − 1 and 1, the ternary NOR gate operations are performed in a similar way to the binary NOR. For Output Logic 0, both p- and n-channel modes are turned on by Input Logic 0. Subsequently, a current path between *V*_DD_ and *V*_SS_ is created and a *V*_OUT_ of approximately 0 V is obtained.

The average energy consumption per logic operation estimated for the binary and ternary logic of the inverter and NAND and NOR gates is 1.43 nJ. When Output Logic 0 is obtained in the ternary logic operation, the average energy consumption is estimated relatively high at 3.49 nJ because the component transistors in both the pull-up and -down networks are turned on. On the other hand, when the logic operations other than Output Logic 0 are performed, the average energy consumption is low at 0.88 nJ because either the pull-up or -down networks is turned on. All of the above energy consumption is caused by the short-circuit current occurring during logic transitions, and there is no energy consumption during the hold operations because the supply voltages are 0 V.

Additionally, as shown in Figs. 4, 5, and 6, binary and ternary logic operations of inverters and NAND and NOR gates comprising NS FBFETs exhibit the voltage mismatch between *V*_IN_ and *V*_OUT_ which becomes a constraint in the logic cascading where *V*_OUT_ is applied to *V*_IN_ in the next stage. The component transistors store the charge carriers in the channel regions, and thereby the voltage degradation occurs in *V*_OUT_ during logic operations, which causes the voltage mismatch. Accordingly, future research includes suggesting solutions to the voltage mismatch problem and demonstrating the logic operations in the logic cascading through experiments or simulations. For example, an experiment can be conducted to eliminate the voltage mismatch by using a voltage amplifier to compensate for the voltage degradation in *V*_OUT_ and applying it to the *V*_IN_ of the next stage.

Notably, each ternary logic operation of our inverters and NAND and NOR gates can be performed by the same circuit as the corresponding binary logic operation, which differs from conventional CMOS logic gate circuits: a CMOS ternary inverting operation requires five transistors and two supply voltages, and the CMOS logic gate operations of ternary NAND and NOR require three more transistors than the corresponding binary logic operations^33^. Our ternary logic gates follow the same topology as CMOS binary logic gates. Hence, they can perform ternary logic operations with fewer transistors than CMOS ternary logic gates, and the decrease in the number of parasitic capacitors increases the operating speed when compared with conventional CMOS logic gate circuits.

Binary and ternary logic gates have been switched in logic systems comprising heterojunction transistors with negative differential transconductance or negative differential resistance characteristics^34–36^. These logic systems perform MVL operations while maintaining the same number of transistors required for logic gate operations as in binary logic owing to the unique characteristics of heterojunction transistors. However, they switch between binary and ternary logic gates through programming stages (such as applying bias and laser irradiation) or operate at high voltages (several tens of volts), which can cause defects in the transistors. Conversely, a logic system consisting of NS FBFETs can perform binary and ternary logic operations in a single-circuit structure, even at relatively low voltages, without programming stages. Thus, NS FBFETs show a promising application potential as components of binary and ternary logic systems.

## Conclusions

In this study, we demonstrated the binary and ternary LIM operations of inverters and NAND and NOR gates comprising NS FBFETs with a triple-gated structure. The NS FBFETs reconfigured in the p- or n-channel modes exhibited excellent switching characteristics, with extremely low *SS* and high *I*_ON_/*I*_OFF_. The fabricated logic circuits comprising NS FBFETs can perform inverter, NAND, and NOR operations. Both binary and ternary logic operations can be performed in each circuit, and these circuits store the outputs even when all external voltages are 0 V. Therefore, our LIM is expected to serve as a bridge between conventional binary logic systems and data processing efficiency-enhanced ternary logic systems. Furthermore, addressing the voltage mismatch issue, which remains our future research for the logic cascading, could lead our LIM to the core technology of next-generation computing.

## Data Availability

All data generated during this study are included in this published article.

## References

[1] Hilbert M, López P (2011). The world’s technological capacity to store, communicate, and compute information. science.

[2] Yang C, Huang Q, Li Z, Liu K, Hu F (2017). Big Data and cloud computing: Innovation opportunities and challenges. Int. J. Digit. Earth.

[3] Ionescu, A. M. in *2017 IEEE International Electron Devices Meeting (IEDM).* 1.2. 1–1.2. 8 (IEEE).

[4] Rydning DR-JG-J, Reinsel J, Gantz J (2018). The digitization of the world from edge to core. Framingham: Int. Data Corp..

[5] Wulf WA, McKee SA (1995). Hitting the memory wall: Implications of the obvious. ACM SIGARCH Comput. Archit. News.

[6] Xu L (2019). Memristor-based efficient in-memory logic for cryptologic and arithmetic applications. Adv. Mater. Technol..

[7] Zou X, Xu S, Chen X, Yan L, Han Y (2021). Breaking the von Neumann bottleneck: architecture-level processing-in-memory technology. SCIENCE CHINA Inf. Sci..

[8] Hou X (2020). A logic-memory transistor with the integration of visible information sensing-memory-processing. Adv. Sci..

[9] Reis, D., Niemier, M. & Hu, X. S. in *Proceedings of the international symposium on low power electronics and design.* 1–6.

[10] Gupta, S., Imani, M. & Rosing, T. in *2018 IEEE/ACM International Conference on Computer-Aided Design (ICCAD).* 1–7 (IEEE).

[11] Mutlu O, Ghose S, Gómez-Luna J, Ausavarungnirun R (2019). Processing data where it makes sense: Enabling in-memory computation. Microprocess. Microsyst..

[12] Wang Z-R (2016). Functionally complete Boolean logic in 1T1R resistive random access memory. IEEE Electron Device Lett..

[13] Wang C, Wang Z, Wang G, Zhang Y, Zhao W (2020). Design of an area-efficient computing in memory platform based on STT-MRAM. IEEE Trans. Magn..

[14] Yin X, Chen X, Niemier M, Hu XS (2018). Ferroelectric FETs-based nonvolatile logic-in-memory circuits. IEEE Tans. Very Large Scale Integr. (VLSI) Syst..

[15] Thirumala, S. K., Jain, S., Raghunathan, A. & Gupta, S. K. in *2019 IEEE/ACM International Symposium on Low Power Electronics and Design (ISLPED).* 1–6 (IEEE).

[16] Hurst (1984). Multiple-valued logic—Its status and its future. IEEE Trans. Comput..

[17] Smith KC (1981). The prospects for multivalued logic: A technology and applications view. IEEE Trans. Comput..

[18] Jeong JW (2019). Tunnelling-based ternary metal–oxide–semiconductor technology. Nature Electronics.

[19] Liu, W., Sun, Y., He, W. & Wang, Q. in *2021 IEEE International Symposium on Circuits and Systems (ISCAS).* 1–5 (IEEE).

[20] Liang J, Chen L, Han J, Lombardi F (2014). Design and evaluation of multiple valued logic gates using pseudo N-type carbon nanotube FETs. IEEE Trans. Nanotechnol..

[21] Sandhie ZT, Patel JA, Ahmed FU, Chowdhury MH (2021). Investigation of multiple-valued logic technologies for beyond-binary era. ACM Comput. Surv. (CSUR).

[22] Eslami N, Moaiyeri MH (2023). A flexible and reliable RRAM-based in-memory computing architecture for data-intensive applications. IEEE Trans. Emerg. Top. Comput..

[23] Gargari MA, Eslami N, Moaiyeri MH (2023). An energy efficient in-memory computing architecture using reconfigurable magnetic logic circuits for big data processing. IEEE Trans. Magn..

[24] Razi F, Moaiyeri MH, Mohammadi S (2021). A magnetic reconfigurable ternary NOR/NAND Logic for logic-in-memory applications. Spin.

[25] Lim D, Son J, Cho K, Kim S (2020). Quasi-nonvolatile silicon memory device. Adv. Mater. Technol..

[26] Kim M (2016). Steep switching characteristics of single-gated feedback field-effect transistors. Nanotechnology.

[27] Kim Y (2018). Switchable-memory operation of silicon nanowire transistor. Adv. Electron. Mater..

[28] Son J, Cho K, Kim S (2022). New ternary inverter with memory function using silicon feedback field-effect transistors. Sci. Rep..

[29] Mikolajick T, Heinzig A, Trommer J, Baldauf T, Weber WM (2017). The RFET—A reconfigurable nanowire transistor and its application to novel electronic circuits and systems. Semicond. Sci. Technol..

[30] Weber W (2014). Reconfigurable nanowire electronics—A review. Solid-State Electron..

[31] Mallik A, Chattopadhyay A (2011). Drain-dependence of tunnel field-effect transistor characteristics: The role of the channel. IEEE Trans. electron Devices.

[32] Gupta G, Rajasekharan B, Hueting RJ (2017). Electrostatic doping in semiconductor devices. IEEE Trans. Electron Devices.

[33] Wu C-Y, Huang H-Y (1993). Design and application of pipelined dynamic CMOS ternary logic and simple ternary differential logic. IEEE J Solid-State Circuits.

[34] Duong NT (2019). Modulating the functions of MoS_2_/MoTe_2_ van der Waals heterostructure via thickness variation. ACS Nano.

[35] Srivastava PK (2019). Multifunctional van der Waals broken-gap heterojunction. Small.

[36] Lee C (2023). A reconfigurable binary/ternary logic conversion-in-memory based on drain-aligned floating-gate heterojunction transistors. Nat. Commun..

